# Paperboard Coating Detection Based on Full-Stokes Imaging Polarimetry

**DOI:** 10.3390/s21010208

**Published:** 2020-12-31

**Authors:** Javier Brugés Martelo, Jan Lundgren, Mattias Andersson

**Affiliations:** 1 Electronic Design Department, Mid Sweden University, 851 70 Sundsvall, Sweden; jan.lundgren@miun.se; 2 Department of Design, Mid Sweden University, 831 25 Örnsköldsvik, Sweden; mattias.andersson@miun.se

**Keywords:** imaging polarimetry, stokes parameters, extruded plastic coatings, support vector machines

## Abstract

The manufacturing of high-quality extruded low-density polyethylene (PE) paperboard intended for the food packaging industry relies on manual, intrusive, and destructive off-line inspection by the process operators to assess the overall quality and functionality of the product. Defects such as cracks, pinholes, and local thickness variations in the coating can occur at any location in the reel, affecting the sealable property of the product. To detect these defects locally, imaging systems must discriminate between the substrate and the coating. We propose an active full-Stokes imaging polarimetry for the classification of the PE-coated paperboard and its substrate (before applying the PE coating) from industrially manufactured samples. The optical system is based on vertically polarized illumination and a novel full-Stokes imaging polarimetry camera system. From the various parameters obtained by polarimetry measurements, we propose implementing feature selection based on the distance correlation statistical method and, subsequently, the implementation of a support vector machine algorithm that uses a nonlinear Gaussian kernel function. Our implementation achieves 99.74% classification accuracy. An imaging polarimetry system with high spatial resolution and pixel-wise metrological characteristics to provide polarization information, capable of material classification, can be used for in-process control of manufacturing coated paperboard.

## 1. Introduction

Extruded and laminated plastic coatings are used on paperboard for food packaging applications. Environmental concerns about the impact of such polymers have increased the need for the development and application of new types of coatings for packaging [1]. However, the low-cost production and excellent barrier properties of plastic coatings on paperboard still outperform those novels’ environmentally friendly solutions. Different types of plastic coatings have been formulated for different packaging end uses [2]. Low-density polyethylene (PE)-coated paperboard is the most widespread plastic-coated paperboard used in the packaging industry due to its good optical and mechanical properties. The barrier functionality and sealability against liquids of PE coating are preferred for many food packaging products. To reduce the environmental impact of plastic coatings used in packaging and to increase the yield of its manufacturing process, sensing technologies for the measurement of material parameters and detection of defects are necessary to move towards an automated in-process control manufacturing.

To obtain the desired functionality, which is linked to overall product quality, paperboard manufacturers rely mainly on off-line characterization. Holes, cracks, and variation in coating thickness are the main defects affecting overall product quality and its barrier properties after the manufacturing process [3]. Off-line characterization offers a quantitative and qualitative analysis of product quality and comprises standardized methods that can be replicated by others. Several standards have been issued to quantify pinhole formation [4,5]. Local coatings defects, for example, the appearance of holes in the surface and thickness variation of the plastic coating, need to be quantified according to the standards under laboratory conditions. The methods require using a coloring ink that penetrates the product through the defects, making them visible and pointing out the possible leak locations. In a recent study, an optical light microscope, a polarized light microscope, and a scanning electron microscope were used to characterize and quantify the defects [6]. The disadvantage of this method of quality control lies in the synchronization between machine parameters and characterization since this method lacks real-time feedback to the process. Demands for higher product quality and competitive products require more in-process metrology for which online sensors are essential to predict or detect defects during the manufacturing process. Pores and cracks are often undesired in packaging, except when they are intended to add breathability to the material for specific applications. Recent sensor developments for quantifying the size and distribution of holes in the material along the production line have been suggested in the literature. The sizes of holes in the coated paperboard can be estimated by correlating the amount of current leaking between cathodes positioned on both sides of the paperboard, which acts as a capacitor [7]. This method is considered non-destructive, but the high voltage applied by the method can increase the initial size of the pores in the packaging. The distribution of holes in the coating can be determined using reflectometry [8], an optical system that uses polarization of light to determine the optical properties of the material. Characteristics such as average surface roughness and the material’s refractive index are found similar between the base layer and its few-microns-thick PE coating. Based on these characteristics, defect detection and classification become challenging when using traditional machine vision systems based on the intensity and/or spectral measurements.

Polarization is one of the main characteristics of light, along with intensity and wavelength. For humans, perceived optical properties are limited to colors and intensities; polarization is only available to us using instruments. Similarly, in many industrial applications, machine vision often relies solely on detecting color or intensity, or both. Polarization in a machine vision application is used to filter out the haze created by surfaces where the specular reflection in the scene hides the desired information. Machine vision has been implemented in manufacturing to aid in-process product quality monitoring, defect inspection, and material classification, but highly specular and transparent materials challenge the accuracy and robustness of the optical method employed [9,10].

In the last decades, there is an increased interest in the use of imaging polarimeters for material classification [11,12,13]. The technique has been employed in remote sensing [14], astronomy [15], aerospace and defence [16], biomedical applications [17,18,19], and industrial manufacturing [20,21]. Polarimeters can provide partial- or full-polarization information about the captured incoming light, depending on the optical elements employed and the imaging system configuration. Industrially, the metrological features of polarization have been restricted to ellipsometry and reflectometry [22]. The systems used are bulky, require a priori knowledge of the sample’s optical properties, and can measure only small areas of the samples [8,23]. Imaging polarimetry offers an alternative for measuring larger areas with high spatial resolution and including radiance values directly related to polarization. The pixel information is presented in the form of the Stokes parameters:

(1) $$\vec{S}={\left(S_{0},S_{1},S_{2},S_{3}\right)}^{T}.$$

The mathematical formulation of the Stokes parameters is convenient because it has several degrees of freedom and can be further computed to obtain complementary parameters related to physical material properties [24], for example, surface roughness, birefringence, and coating thickness. Stokes parameters are expressed in power reflectance units, like pixels in a commercial camera system record intensity values. Different configurations of imaging polarimeters found in the literature serve specific metrological purposes [20] and are classified according to the temporal or spatial recording of the scene. The advent of new camera systems with embedded micro-polarizer arrays in the division-of-focal-plane configuration offers the possibility of a new compact camera system for machine vision applications in industrial manufacturing processes. Linear imaging polarimeters provide information from the first three Stokes parameters, that is, intensity ($S_{0}$) and linear polarization component ($S_{1}\;,\;S_{2}$), which can be in certain cases sufficient for the industrial application. To measure the complete polarization cues in a scene, a fourth component, that is, the circular polarization component ($S_{3}$), must be acquired. In certain applications in which material properties are similar in terms of surface roughness and spectral information, measuring the complete polarization from the material reflection is desired; for example, in coating applications [25], instantaneous active imaging polarimetry has been employed for measuring the polarization properties of the material. In certain applications in which material properties are similar in terms of surface roughness and spectral information, measuring the complete polarization properties from the material’s light reflection is desired; for example, in paper coating applications [25], different fillers and binders were applied to the paper, and instantaneous active imaging polarimetry has been employed for measuring the polarization properties of the material. In this study, no transparent or thin-film was used to coat the paper material. From our study, we measured the polarization properties of the PE-coated paperboard and its substrate, that is, the paperboard before applying the PE-coating. Instead of using a point-measurement system, we proposed used a pixelated camera system that can obtain the full polarization properties of the material with high-spatial-resolution. An image containing a set of polarization cues or features represented in the form of Stokes parameters are obtained, and can directly relate to the optical properties of each material. These features can then be used as predictors of whether or not there exists a PE-coating on the paperboard. Then, we use a supervised machine learning algorithm, that is, support vector machine (SVM), for classification based on these polarimetric features. SVM [26] is a set of classification and regression algorithms widely adopted in industrial applications. For the classification task, SVM requires to modify a few set of parameters to balance the performance and accuracy of the result. In the context of non-linearly separable data, a kernel-function (e.g., polynomial or basis radial function) might transform the non-linear problem into a linear problem, returning the maximal separable margin that divides the classes.

In this article, we present an active full-Stokes imaging polarimeter capable of detecting the extruded PE coating on high-quality paperboard with high spatial resolution and pixel-wise polarization metrology. The optical system is based on full-Stokes imaging polarimetry and controlled polarized white-light LED illumination. Generalizing the classification technique proves that the system can discern the presence or absence of the extruded coating on the paperboard. In our understanding, the classification solution presented can be further exploited for detecting and quantifying defects such as pinholes and cracks in large areas of paperboard manufactured for packaging.

## 2. Material and Methods

We summarize our strategy in three main parts as follows:

1. polarimetric measurement of PE- and non-PE coated paperboard samples using the full-Stokes imaging polarimeter, data acquisition, and polarimeter parameter calculations;
2. implementation of the distance correlation function for the feature selection of the polarimetric measurements; and
3. model training and validation for material classification using supervised learning algorithms, that is, support vector machine (SVM) algorithms.

**Figure 1:**
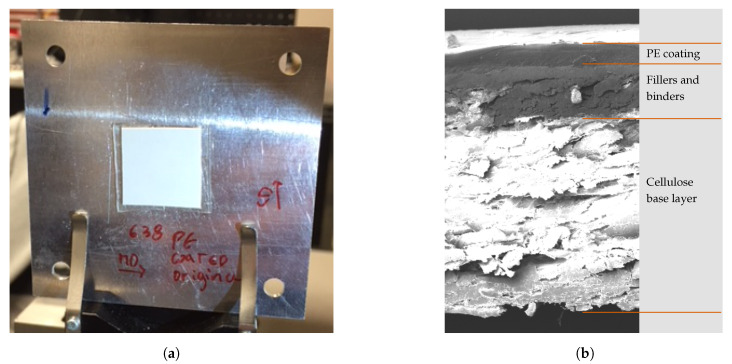
(**a**) adapted 20 × 20 mm sample illuminated by the optical system. (**b**) scanning electron microscope (SEM) crosssection image, (working distance 10-mm, field-of-view 200-µm), of PE-coated paperboard. The paperboard is composed of a multi-ply cellulose material, a coating layer of binders and fillers, and a top PE-coating layer with an average thickness of 13 µm.

Industrially manufactured paperboard samples were obtained from a local paperboard manufacturer for use in this study. Before and after the extruded low-density polyethylene (PE) coating was applied on one side of the paperboard, A4 sheet-sized samples were extracted from the manufacturing process, resulting in paired samples from the same process. The paperboard is multi-ply in structure, consisting of a cellulose base layer coated with a mixture of binders and fillers on the side of the PE coating before the PE coating is applied. This mixture of binders and fillers is added to the cellulose material to increase the smoothness of the multi-ply structure, ensuring good adhesion of the PE coating and resulting in a more even coating thickness. This mixture also reduces the effects of undesired protruding cellulose fibres that can puncture the extruded coating layer and cause cracks, pinholes, and local inconsistencies in the PE thickness. These defects decrease the overall quality of packaging material, degrading its most important properties as a fat, oil, and moisture barrier [2]. We removed 20 × 20-mm pieces from the original samples, placing them in a sample holder as shown in Figure 1a to make measurements using the imaging polarimeter. The average thickness of the PE coating layer was 13 μm; in Figure 1b this layer is shown in a PE-coated sample cross-section as imaged using a scanning electron microscope (SEM) (TESCAN MAIA3 GMU 164 manufactured by TESCAN Brno, s.r.o.; Brno, Czech Republic).

**Figure 2:**
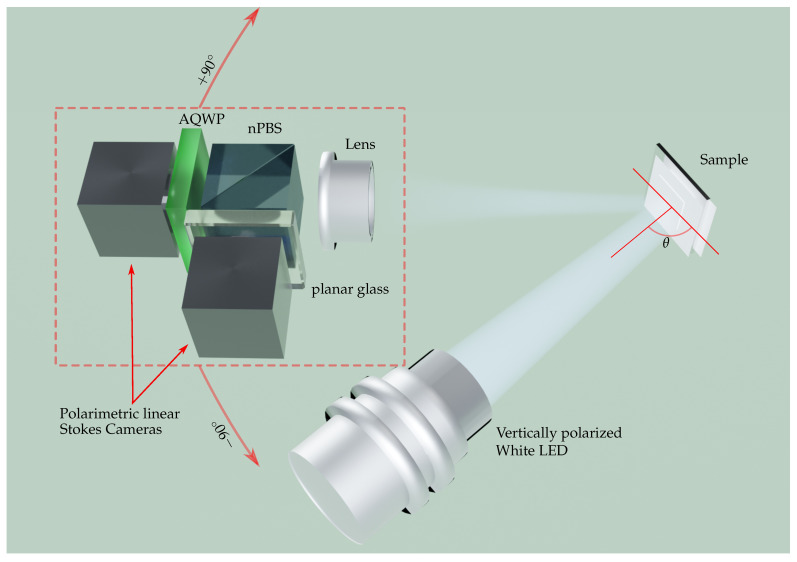
Active full-Stokes imaging polarimetry.

Figure 2 shows the experimental set-up. An active imaging polarimeter measures the sample at an oblique angle while a high-luminosity white LED (Thorlabs, Inc. Ref. MWWHL4, 570 mW and spectral range 400–700 nm) illuminates it with vertically polarized light. The sensor in the active imaging polarimeter is based on a novel full-Stokes imaging polarimetry camera system constructed by the Imaging and Applied Optics Lab—Prof. R. Liang’s group—at the School of Optical Science, University of Arizona. For a complete description of the full-Stokes imaging polarimeter, the authors refer the reader to Tu et al. [27], who provides details of the design, the calibration, and the demosaicing algorithm. A brief description of the optical system’s components is presented here. Two linear polarimetric cameras in a division-of-amplitude configuration are employed as the sensors of the system, in which two balanced optical paths with complementary polarization information are divided from the incoming light. This path division of the light allows the instantaneous measurement of the complete polarization. An imaging lens focusing on the measured surface is placed in front of the polarimeter assembly. A 50/50 non-polarizing beam-splitter (nPBS) divides the incident beam into two balanced paths. As mentioned, two complementary polarization measurements are required to obtain the full-Stokes vector. In one arm, an achromatic quarter-wave plate (AQWP) in the visible spectral range will divide the beam into its circularly polarized components. In the second arm, to balance the path difference created by the AQWP, a planar glass with a similar thickness as the AQWP is placed. Finally, in both arms, linear Stokes polarimetric cameras capture the scene. With the appropriate demosaicing and calibration algorithms, the calibration and synchronization of the cameras make it possible to obtain pixel-wise polarization and spectral radiance information on the scene in a single shot.

**Figure 3:**
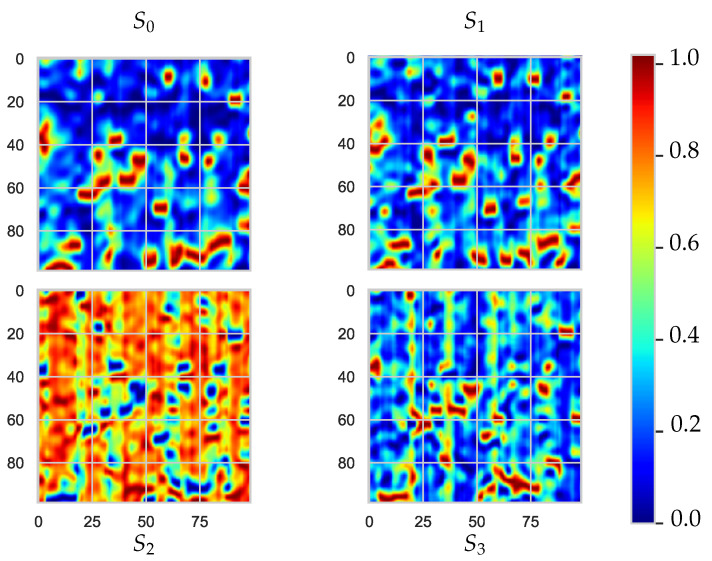
Polyethylene (PE)-coated sample polarization measurement: $S_{0}$, $S_{1}$, $S_{2}$ and $S_{3}$ are the Stokes vector as captured by full-Stokes imaging polarimetry.

Figure 3 shows the PE-coated paperboard sample image of each Stokes parameter after being measured by the active full-Stokes imaging polarimeter. The 20×20-mm sample is resolved with a spatial resolution of 37 µm in a single pixel. The on-axis set-up shown in Figure 2 was built to rotate the camera system around the sample, and illumination was fixed at 45 degrees with respect to the sample’s surface normal. Several in-plane measurements were made at different angles around the sample in 5-degree steps. Before the parameter selection for the experiment, we analysed all the different positions to determine the best angle configuration. It was found that the best angle between the camera and the normal to the sample surface was 40 and 55 degrees, that is, around the specular direction. In dielectrics, when illuminating with vertically polarized light, roughness increases greatly affect the angles of polarization due to the retardation of the light wave phase component [28].

As opposed to passive imaging techniques in which the polarization state of the illumination is uncontrolled, in an active polarimeter, the polarization state of the incident light is known. In our proposed set-up, the illumination is vertically polarized. Then, the beam reflected by the samples is either partially polarized or completely depolarized, in contrast to the incident light. The sensor in our polarimeter is based on a full-Stokes imaging system, which can obtain in one shot pixel-wise information in the form of the Stokes vector (1). At dielectrics and interfaces, the polarization of a beam undergoes different phase delays resulting in changes in polarization.

In imaging polarimetry systems used in remote sensing and biomedical applications, the features used in algorithms for material classification are parameters calculated from the measured Stokes vector. These polarimetric parameters are related to the material’s optical properties and can be easily mathematically formulated. We have calculated three parameters from the initial Stokes vector, that is, the degree of polarization (DoP), *p*, the azimuth, φ, and the ellipticity, χ, and we first evaluated their statistical significance for the classification task using a distance correlation function. The DoP, *p*, is expressed as

(2) $$p=\frac{\sqrt{{S_{1}}^{2}+{S_{2}}^{2}+{S_{3}}^{2}}}{S_{0}},$$

which is inversely related to the depolarization effect, that is, the ratio at which polarized light is partially polarized or unpolarized by the material under polarized illumination. Using the three last components of the Stokes vector-like axis in Cartesian coordinate space and representing the polarization measurements inside this coordinate space, angular relations can be derived. Two angles can be calculated, the azimuth, φ, and the ellipticity, χ, as

(3) $$\chi \;=\tan\left(\frac{1}{2}\arcsin\left(\frac{S_{3}^{2}}{\sqrt{S_{1}^{2}+S_{2}^{2}+S_{3}^{2}}}\right)\right)\;,\;-\pi /4\leq \;\chi \;\leq \;+\pi /4,$$

(4) $$\varphi \;=\;\frac{1}{2}\arctan\left(\frac{S_{2}}{S_{1}}\right)\;,\;0\leq \;\varphi \;<\;\pi ,$$

where χ, at the extremes of the inequality represents a fully circularly polarized beam when points in the sphere are located at either side of the poles; χ=0 being linearly polarized otherwise the beam is elliptically polarized. The azimuth angle, φ, is often described in the literature as the angle of linear polarization (AoLP). It measures the linear relation of the observed beam and concerns only the linear polarization components of the Stokes vector in the equation.

**Figure 4:**
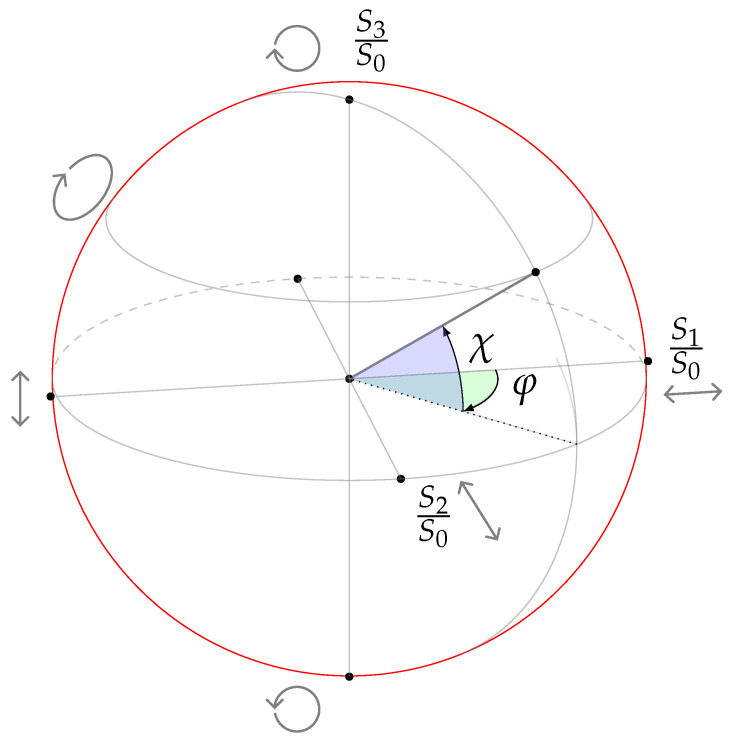
Observable Poincaré sphere of polarization states. Associated with any point on the sphere is a unique polarization state described either by spherical angular coordinates (φ, χ) or normalized Stokes parameters $S_{1}/S_{0}$, $S_{2}/S_{0}$ and $S_{3}/S_{0}$.

**Figure 5:**
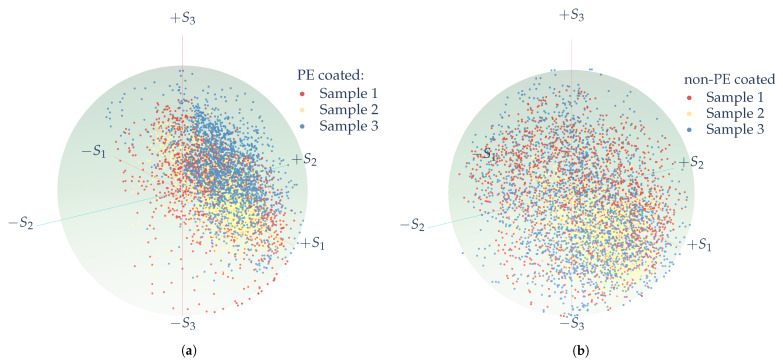
Poincaré unit sphere representation from imaging polarimetry measurements of (**a**) PE-coated paperboard and (**b**) non-PE coated paperboard. Each pixel value was normalized by its *S*_0_ pixel value, and three polarimetric measurements of each class are presented.

A Poincaré sphere, first presented by Henri Poincaré (1892), helps visualize these angular relations (see Figure 4). Figure 5 shows the Poincaré spheres for PE- and non-PE coated paperboard samples. In this figure, each sphere represents three measurements for each class. Pixel-wise information in the form of the Stokes vector (1) are obtained in each measurement and then the vector is normalized concerning the intensity value of $S_{0}$. In Figure 5, each point in the surface of the sphere represents the normalized Stokes vector, resulting in a unit Poincaré sphere. The angular distributions of the points on the surface of the sphere differ due to the unique optical properties of the sampled material and the angles, φ and χ, from the spherical coordinates system, are used as preliminary features for the material classification.

We implemented a distance correlation metric, in which we look for the pair of features ranking the highest and finally provided the best accuracy for the classification. Sample correlation functions are employed to measure the linear and nonlinear relations between variables. Distance correlation functions based on measurements of the correlation between the features are employed to rank the importance of the features. The distance correlation function presented by Szekely et al. [29] has been demonstrated to be useful in feature selection for relatively small datasets with high dimensionality [30]. This metric does not require any *a priori* knowledge of the feature distribution, and it has advantages over more commonly used statistical tests, such as Pearson correlation, which cannot detect nonlinear relations between the evaluated variables. A summary of the implementation according to Reference [29] follows. We first obtain the defined distance covariance for each combination of features,

(5) $$dCov\left(Fx,Fy\right)=\frac{1}{n^{2}}\sum_{i=1}^{n}\sum_{j=1}^{n}D\left(fx_{i},fx_{j}\right)\cdot D\left(fy_{i},fy_{j}\right),$$

where $D\left(fx_{i},fx_{j}\right)=∥fx_{i}\;-\;fx_{j}∥,\;\mathrm{and}\;D\left(fy_{i},fy_{j}\right)=∥fy_{i}\;-\;fy_{j}∥$, are the centered Euclidean distances of the feature vectors FxandFy with scalar values fxandfy, respectively. Finally, we can calculate the distance correlation *(dCor)*,

(6) $$dCor\left(Fx,Fy\right)\;=\frac{\;dCov(Fx,Fy)}{\sqrt{\nu_{Fx}^{2}\cdot \nu_{Fy}^{2}}},$$

where $\nu_{Fx}^{2}\;\mathrm{and}\;\nu_{Fy}^{2}$ are the positive feature variances. If FxandFy are independent features, then the *(dCor)* will be equal zero. We are interested in features with the largest distance correlation, as they benefit the class separation and classification accuracy of the supervised learning algorithm. The result is the selection of the best features, which is meant to be a preprocessing step in our machine learning approach. This reduces the computational cost without compromising the accuracy of the resulting classification model. We have implemented this algorithm to rank and select the best pair of features for the classification algorithm described in our machine learning pipeline.

### 2.1. SVM Algorithm

For the classification process, we implemented a support vector machine (SVM) algorithm with the use of a Gaussian kernel function using selected features of the two sample classes, that is, PE- and non-PE coated paperboard. SVM algorithms have proven to be robust in implementation for industrial classification problems. When the features in the dataset present non-linear relations, the SVM algorithm can use kernel functions to find the maximum separable hyperplane for classification. Gaussian and polynomial kernel functions are an example of nonlinear kernel functions. The kernel function will map the nonlinear data from the original dimensional space into a higher-dimensional space. This may result in a linearly separable problem where a hyperplane can divide the classes with the maximum margin possible. In this experiment, we constructed the SVM model in Python using scikit-learn libraries for supervised learning based on the library for support vector machines (LIBSVM).

### 2.2. SVM Algorithm Pipeline

**Figure 6:**
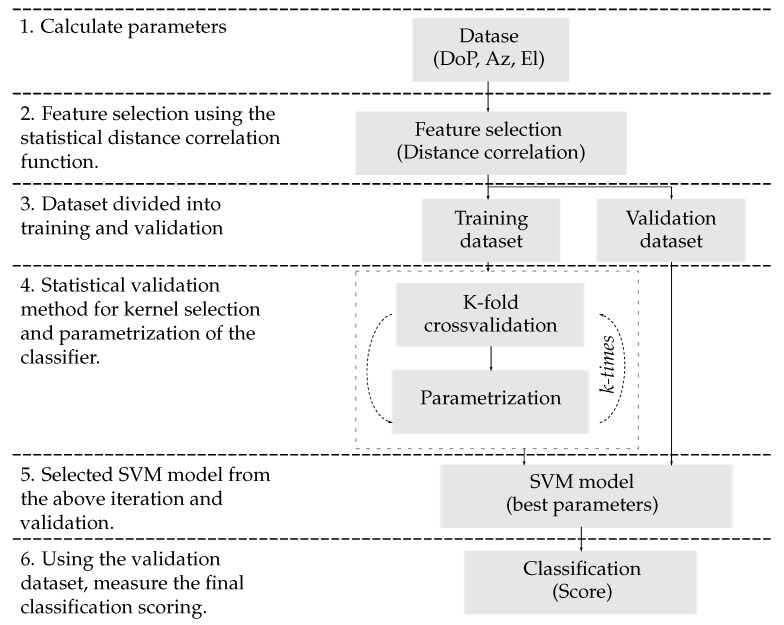
Workflow pipeline for the training, validation and selection of the classification algorithm.

We obtained 24 polarimetric measurements for both classes (i.e., 12 polarimetric measurements each for the PE- and non-PE coated paperboard) included in the dataset. The measurements were taken from different regions of the initial industrial samples. Each measurement contains 400 × 70 pixels with the individual values of the Stokes vectors. We calculated the polarimetric features at each pixel, as described in the previous section. The pipeline of our classification approach is described in Figure 6. After obtaining the data, we implemented a feature selection-based distance correlation function (*dCorr*) (6), selecting the two best-ranked features. We then partitioned in a 50–50 split of the dataset for training and validation. Because of dealing with a limited number of samples, it is appropriate to determine the best possible combination for training and validation without degrading performance or causing underfitting. We observed that by increasing the data for training and when reaching 50% of the complete dataset, there is no significant increase in the score in either model. We presume that the simplicity in identifying the classes relies on the strong correlation between the polarimetric features at each pixel in the recorded images and the surface homogeneity resulted from the high quality of the samples.

We contrasted two nonlinear kernel functions for SVM, that is, a Gaussian and a second-degree polynomial kernel function based on statistical significance test from the classification accuracy after training and validation. To obtain the statistical significance of the resulting model, we used 10-fold cross-validation since SVM does not account for statistical score interpretation. We conducted the cross-validation twice. The first time was to obtain the best parameter combination for each kernel. Then, we repeated 10-fold cross-validation, in this case on each parameterized kernel function, and selecting the one with the best performance. Finally, to test the generalization of the model, we performed the classification of PE- and non-PE coated paperboard using a polarimetry measurement that was not included in the dataset used to train the classifier.

## 3. Results and Discussions

### 3.1. Feature Selection

Pixel-wise polarimetric values of the degree of polarization (dop) (2), and azimuth (az) (4), and ellipticity (el) (3) angles were calculated from the measured Stokes parameters.

**Figure 7:**
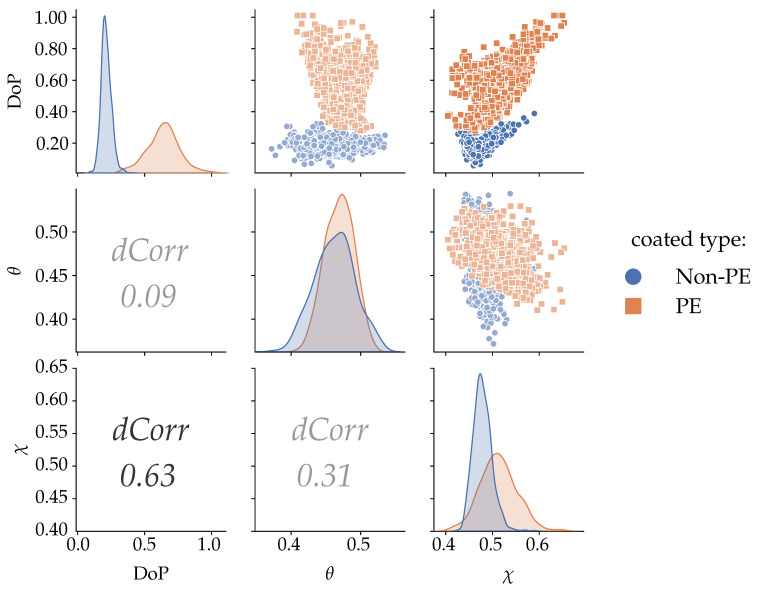
Scatter matrix displaying the relation between features degree of polarization (DoP), φ, and χ. The *dCorr* value represents the distance correlation between the features. All the features in the dataset are normalized and only 20% of the dataset is shown to aid in the visualization.

We evaluated the distance correlation between these three independent parameters to assess the best of these parameters for the classification problem. In the feature selection step, we measured the correlation between these polarization parameters. The metric for the distance correlation function (*dCorr* (6)) is based on the distance covariance (*dCov* (5)) and was used as a feature selection tool. Figure 7 shows the pairwise relations among these three parameters, that is, DoP and the angular variables θandχ, plotted in the form of a scatter matrix. The diagonal of the matrix represents the parameter distribution, the top corner shows the correlation between features in the form of a scatterplot, and the bottom corner shows the value of the distance correlation (*dCorr*) function for each pair of features.

From the scatter matrix, the combination of DoP and the angular parameters could be tested for classification, while the angles themselves displayed considerable overlapping. The relation between DoP and χ scored the highest in distance correlation, suggesting to be the best candidates for classifying PE- and non-PE-coated paperboard, and which we further selected for training the classifier.

### 3.2. Classifiers Parametrization, Training and Validation

**Figure 8:**
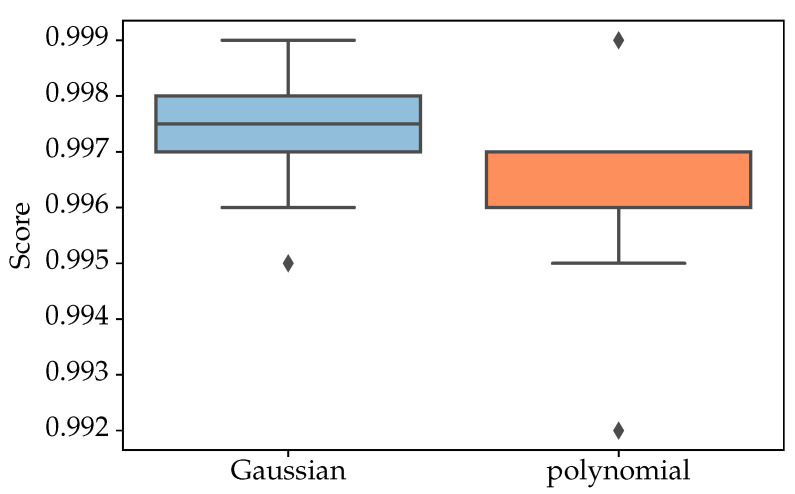
Support Vector Machine (SVM Gaussian and second-degree polynomial kernel function accuracy scores after 10-fold cross-validation.

We next evaluate the classification performance of two SVM kernel functions, that is, Gaussian and second-degree polynomial kernel functions. Before comparing the kernels’ classification’s scores, it is necessary to find the best set of parameters for each kernel. For each kernel, a set of predefined parameters is tested to determine the classification score and measure the recall of the model, that is, the true positives the model has correctly obtained as a percentage of all positive cases, so we can select the best combination. After applying the cross-validation, the Gaussian kernel with the hyperparameters C = 100 and γ= 0.1 and the second-degree polynomial with the hyperparameter C = 100 were selected. With these specified parameters, we proceed to evaluate and compare the scoring accuracy of both models. Figure 8 shows the difference in statistical significance between the two kernels after using 10-fold cross-validation with the selected hyperparameters for each kernel function. We obtained a ratio of about 99.74 ± 0.12% for the Gaussian and 99.61 ± 0.16% for the polynomial degree 2 kernel functions, suggesting a smaller variation and higher score of the former after cross-validation.

**Figure 9:**
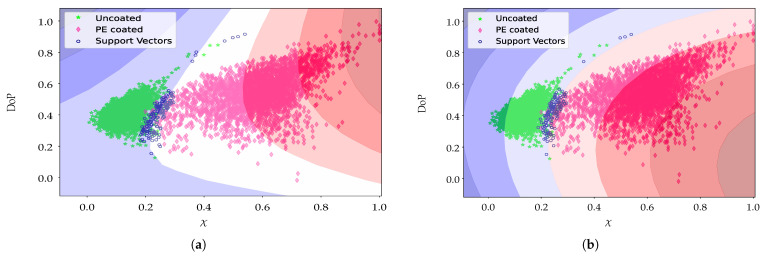
Feature space representation of the classification model of the binary classes PE- and non-PE-coated paperboard implemented with an (**a**) polynomial degree two and (**b**) SVM Gaussian kernel classifier.

Based on the previous results, we trained the model using the complete training dataset. Figure 9 shows the classification and class division by the model’s hyperplane in the original space, where observes an area of overlap between the classes and the support vectors that will lead to misclassification. However, a good generalization for the classification is achieved due to the robust relationship of the polarimetric parameters with the expected classes and the non-linear curved separation found by the SVM kernels. Based on the parameter distribution the Gaussian kernel provides better accuracy for the classification than the polynomial classifier.

**Figure 10:**
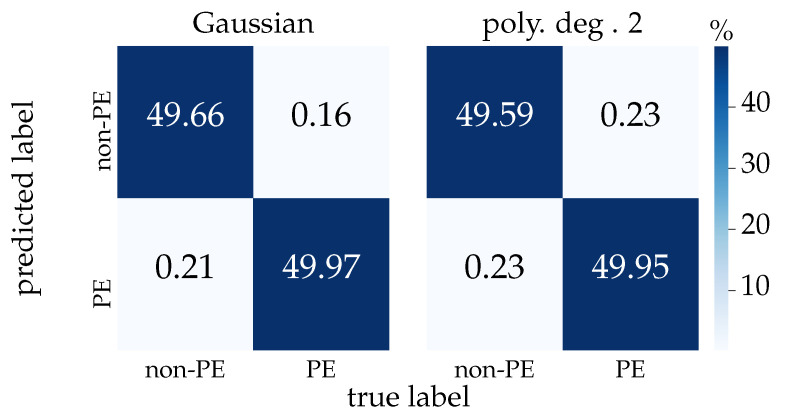
SVM Gaussian and second-degree polynomial kernel function confusion matrix using the validation dataset on the trained support vector classifiers.

We finally used the validation dataset on the resulting models of both classifiers to compare their prediction accuracy. Figure 10 shows the confusion matrix after using the validation dataset. We can observe that a better performance is achieved by the Gaussian kernel as expected from the cross-validation results.

### 3.3. Classifier Testing

**Figure 11:**
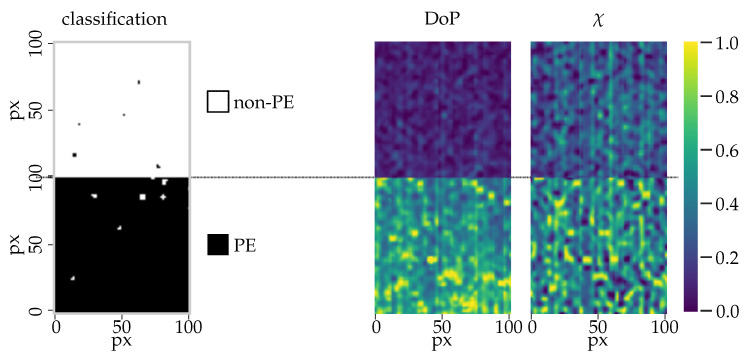
Test dataset with non-PE- and PE-coated paperboard samples (left–right): model classification, DoP, and χ. The values of the features have been normalized to a range of 0 to 1.

In the previous section, a cross-validation test provided both statistical significances for the training and validation, while a validation dataset was used to test the accuracy of the resulting models. We now test the classification model using a new set of data from two new samples, that is, PE- and non-PE coated paperboard. The algorithm can separate the PE-coated from the non-PE coated paperboard, indicating the robustness of the method with almost no misclassification as we can observe in Figure 11. The misclassified areas in both results can be associated with physical properties, like local variations in the topography of the samples or imperfections within the PE coating, which may required further investigation to associate them with the changes in the polarization parameters.

To extend the discussion of whether these areas can be regarded as misclassified, further experiments are required. In the case of PE-coated areas, the scattering mechanism within the coating and reflection from the base layer can affect the accuracy of the measurement. It is also possible that subsurface scattering from irregularities or defects within the extruded material may generate these effects. In the case of the non-PE coated material, misclassification can result from geometric constraints, areas with abrupt changes in the surface roughness, or defects in the mixture coating. This analysis could be performed in a later study, in which our understanding of the optical properties of each material could be extended using complementary techniques and instrumentation. However, for large-area manufacturing processes of high-quality products, in which over 99% of the pixels measured from the samples are of the expected class, the areas where misclassification occurs can be tracked and investigated using current off-line production methods.

Potentially, this system could eliminate some of the off-line quality-control practices in the manufacturing of paperboard by instead using quantitative polarimetric imaging systems for the in-process control, where defects such as pinhole and cracks, found in the coating need to be monitored.

## 4. Conclusions

We proposed using active imaging polarimetry to classify industrially manufactured polyethylene (PE)-coated paperboard and its substrate. With high spatial resolution, instantaneous acquisition, and pixel-wise metrological polarization information, we derived a set of polarimetric features, that is, degree of polarization (DoP), ellipticity, χ, and azimuth, φ, which were later analysed in the classification application. We proposed a robust feature selection method based on distance correlation to reduce the computational cost of the algorithm while not compromising the classification accuracy. From the selected features, we implemented a support vector machine (SVM) classifier that uses a nonlinear kernel trick, that is, a Gaussian kernel function, obtaining 99.7% classification accuracy. We demonstrated active polarimetry based on a full-Stokes imaging system with vertically polarized illumination of the sample for in-process metrology, in which complete polarization information provides a set of robust features directly related to the material’s optical properties that are undetectable by other machine vision systems. To the author’s knowledge, no such study or implementation has so far been proposed.

In future work, we will explore the relation between spectral information and the polarization information that can be important in the surface characterization of paper-based materials and it is available from the active full-Stokes imaging polarimetry. This could increase the likelihood of finding new correlations for surface parameters or, in the case of quality control, other types of defects originating along the production line.
